# INC-Seq: accurate single molecule reads using nanopore sequencing

**DOI:** 10.1186/s13742-016-0140-7

**Published:** 2016-08-02

**Authors:** Chenhao Li, Kern Rei Chng, Esther Jia Hui Boey, Amanda Hui Qi Ng, Andreas Wilm, Niranjan Nagarajan

**Affiliations:** 1 Genome Institute of Singapore, Singapore, 138672 Singapore; 2 Department of Computer Science, National University of Singapore, Singapore, 117417 Singapore

**Keywords:** Nanopore sequencing, Rolling circle amplification, Barcode sequencing, Consensus algorithms

## Abstract

**Background:**

Nanopore sequencing provides a rapid, cheap and portable real-time sequencing platform with the potential to revolutionize genomics. However, several applications are limited by relatively high single-read error rates (>10 %), including RNA-seq, haplotype sequencing and 16S sequencing.

**Results:**

We developed the Intramolecular-ligated Nanopore Consensus Sequencing (INC-Seq) as a strategy for obtaining long and accurate nanopore reads, starting with low input DNA. Applying INC-Seq for 16S rRNA-based bacterial profiling generated full-length amplicon sequences with a median accuracy >97 %.

**Conclusions:**

INC-Seq reads enabled accurate species-level classification, identification of species at 0.1 % abundance and robust quantification of relative abundances, providing a cheap and effective approach for pathogen detection and microbiome profiling on the MinION system.

**Electronic supplementary material:**

The online version of this article (doi:10.1186/s13742-016-0140-7) contains supplementary material, which is available to authorized users.

## Background

The MinION sequencing platform introduced by Oxford Nanopore Technologies (ONT) is a portable and inexpensive device that generates long reads in real time and can thus further democratize genomics [1, 2]. Its compact form factor and portability, low establishment (about $1,000) and maintenance cost, and speed of analysis make it an attractive option in a range of new settings [2], despite the platform having a comparatively lower throughput and higher cost per base when compared with other sequencing technologies such as Illumina and Pacific Biosciences (PacBio). Compared with short accurate reads on second-generation sequencing platforms (for example, Illumina and Ion Torrent), nanopore sequencing shares the feature of other third-generation platforms, as it trades off base-level accuracy for much longer reads. However, several genomics applications ideally require both long and highly accurate reads (for example, RNA-seq [3], haplotype sequencing [4] and 16S sequencing [5]) to help to discriminate between very similar DNA sequences.

Raw single-strand reads (1D reads) on the MinION platform have been reported to have an error rate >20 %, but more accurate reads (10-15 % median error rate) are typically generated by a protocol that reads the complementary strand and computes an *in silico* consensus (2D reads) [6]. Similar to this idea, experimental protocols have been developed on other sequencing platforms to boost read accuracy by repeatedly sequencing the same parent template molecule, which generates more reliable consensus reads [7–10]. For example, Barcode-directed Assembly for Extra-long Sequencing (BAsE-Seq) [7] uses a sophisticated library preparation protocol to tag each template with a unique barcode, resolving short barcodes with one end of a paired-end Illumina read, and using the other end to reconstruct a consensus template read. PacBio circular consensus sequencing (CCS) [8] (also known as single molecule, real-time (SMRT) CCS) reads through circularized templates repeatedly by synthesis but cannot be directly applied to nanopore sequencing that requires a single stranded linear template to be threaded through the nanopore channel. Circ-Seq [9, 10] generates linear templates (3 repeating units on average) with a protocol that is customized for short templates to be sequenced on the Illumina short-read platform.

We developed Intramolecular-ligated Nanopore Consensus Sequencing (INC-Seq) that uses rolling circle amplification (RCA) of circularized templates to generate linear products (with tandem copies of the template) that can be sequenced on the nanopore platform. Circularization, amplification and nanopore sequencing face several challenges, including (i) generation of hybrid molecules (chimeras), (ii) introduction of polymerase errors, (iii) amplification bias from exponential amplification, and (iv) input DNA requirements of the nanopore platform. These issues are addressed in the experimental protocol of INC-Seq, which allows for the construction of consensus single-molecule reads with a median accuracy >97 %, using low-input DNA. Applying INC-Seq to rRNA-based community profiling allowed for accurate classification at the species level and identification of rare species in the community. INC-Seq profiles were found to be robust and well correlated with known abundances. As 16S rRNA sequencing is often the method of choice for samples with high non-microbial contamination, INC-Seq provides an effective approach for pathogen detection and microbiome profiling that capitalizes on the strengths of the MinION system. In addition, INC-Seq provides an avenue for the use of nanopore sequencing in many genomic applications that require high sequencing accuracy.

**Fig. 1:**
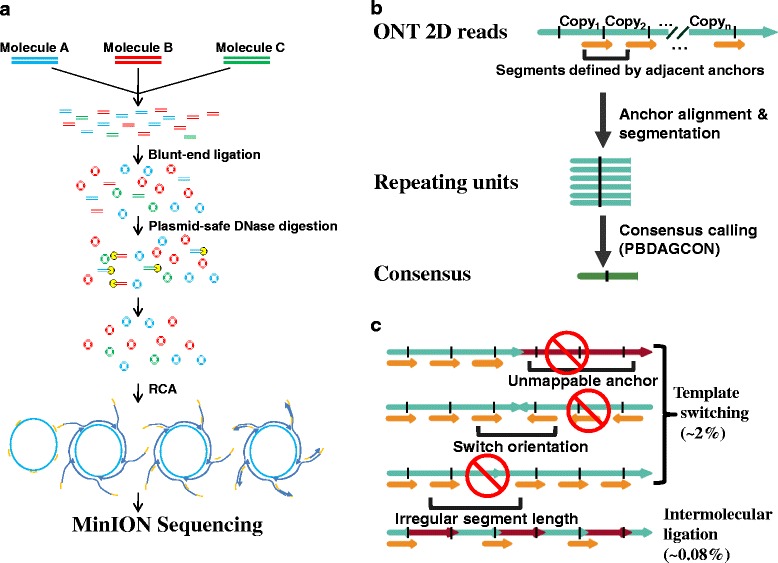
Overview of the INC-Seq workflow. **a** Template molecules are circularized in optimized conditions, and the remaining linear molecules are removed. The circular products are amplified with RCA and sequenced on the MinION platform. **b** A subsequence from the raw nanopore reads is used as an anchor to scan the entire read for the location of repeating units. The repeating units flanked by adjacent anchor starting points are aligned, and a consensus sequence is constructed. **c** In INC-Seq library preparation, chimeras are expected through intermolecular ligation and template switching. Chimeras from template switching are likely to be detected by the anchor mapping protocol. Chimeras from intermolecular ligation were observed to be rare under the experimental conditions used in INC-Seq

### Data description

Statistics for all datasets generated in this study can be found in Table 1. Simulated datasets were generated to evaluate the performance of raw ONT 2D and INC-Seq reads for classification of 16S sequences. Simulated reads were also used to estimate the proportion of chimeras in INC-Seq consensus reads. A ‘simple’ synthetic community with three bacteria was sequenced on the PacBio and MinION platforms to evaluate chimera rates and error rates for the INC-Seq protocol. The ‘ladder’ synthetic community with ten bacteria was sequenced on the MinION platform with two independent replicates to demonstrate the feasibility of applying INC-Seq for 16S sequencing.

**Table 1:** Statistics for sequenced datasets

Dataset	Number of species	Sequencing platform	Statistics before correction			Statistics after correction			Estimated chimera rate	
			Number of 2D pass reads (# of reads)	Number of bases	N50 (average read length)	Number of reads	Number of bases	N50 (average read length)	Template switching	Intermolecular ligation
Simple synthetic community (PacBio)	3	PacBio	- (126456)	669259255	7308 (5292)	16327	11855990	734 (726)	0.03	0.0006
Simple synthetic community (Nanopore)	3	MinION	14583 (45268)	82074997	6807 (5628)	2177	1592303	731 (730)	0.006	0.0009
Ladder synthetic community (replicate 1)	10	MinION	7444 (27937)	31866337	4570 (4280)	1076	794358	739 (738)	-	-
Ladder synthetic community (replicate 2)	10	MinION	2904 (7989)	22446690	10536 (7729)	1183	867494	732 (733)	-	-

## Results

### The INC-Seq workflow

The INC-Seq workflow is based on template circularization, RCA, nanopore sequencing and *in silico* consensus construction (Fig. 1). The experimental procedure for generating INC-Seq libraries is summarized in Fig. 1a. Briefly, ligation conditions were optimized for intramolecular ligation of long DNA molecules to generate predominantly self-ligated circular DNA templates. Non-circular DNA molecules are removed from the ligation mixture by exonuclease treatment. Following this, RCA is carried out to produce long DNA molecules comprising of multiple repeating units. These long DNA products are then gently sheared and prepared for nanopore sequencing.

To correct raw ONT reads, we designed a bioinformatics pipeline that extracts repeating sequence segments and corrects them by constructing a consensus sequence (see ‘Anchor-based consensus construction for INC-Seq reads’ for details; Fig. 1b). For each long read, an anchor subsequence of 500 bp is selected and aligned to the read. Regions between two adjacent anchors define the repeating segments. These repeating segments are aligned to construct a consensus INC-Seq read.

The correction protocol in INC-Seq requires that each read consists of tandem repeats of the same template, and the presence of chimeric repeats can lead to a poor or chimeric consensus. Chimeras can be formed in the INC-Seq protocol owing to intermolecular ligation (during circularization) or to template switching (during RCA). Template switching results in discordant mapping of an anchor, such as an unmappable anchor, orientation switch or irregular anchor distance (Fig. 1c). Chimeras from template switching can thus be detected from the concordance of mapped anchors, except when switching happens between similar templates while preserving orientation and segment length. Chimeras from intermolecular ligation are not directly discovered by the pipeline but generate consensus sequences that are more than twice as long as expected. Chimeras from intermolecular ligation were also observed to be rare under the experimental conditions that are used in INC-Seq (see ‘Assessing chimera rates in INC-Seq libraries with PacBio sequencing’) and can be reduced further by carrying out ligations in more dilute conditions (Fig. 1c).

### Preliminary evaluation of the INC-Seq protocol with simulated reads

**Fig. 2:**
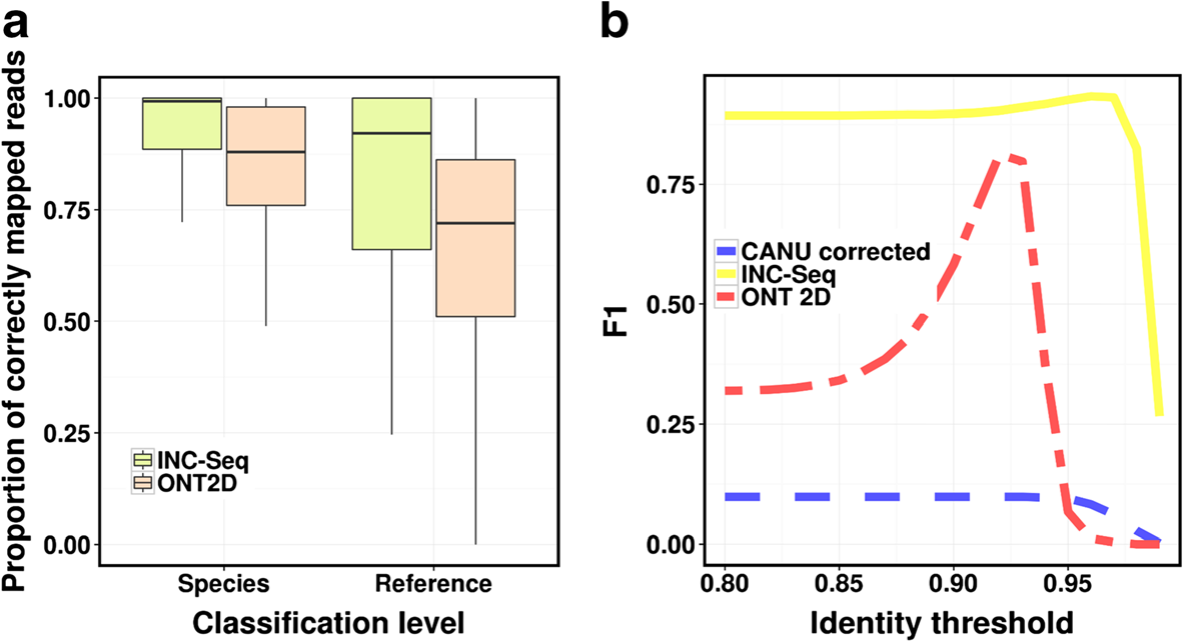
INC-Seq evaluation with simulated reads. **a** 100 representative reference sequences were selected from a customized database. Simulated reads were generated for each reference sequence, as well as artificial RCA products (that is, INC-Seq libraries). Synthetic INC-Seq reads significantly improved the proportion of correctly mapped reads over raw ONT 2D reads at both species and reference level (*p*-value <10^-9^; one-sided paired Wilxcon test). **b** Species-level classification F1 score (that is, the harmonic mean of precision and recall) using simulated raw, CANU corrected and INC-Seq reads from 100 reference sequences and with different identity thresholds for classification (each curve represents the average across 10 replicates)

As a proof of concept, INC-Seq was applied to 16S rRNA profiling owing to its widespread use for bacterial identification, particularly in complex communities and samples with high non-microbial contamination. 16S sequences can be highly similar, and thus both long and accurate reads are ideal for specific taxonomic classification. We began with a preliminary investigation using synthetic reads, as they allow a more extensive investigation of performance. Specifically, synthetic INC-Seq reads were compared with raw 2D reads for their overall ability to accurately identify 16S sequences. Despite the presence of the correct reference sequence in the database, only 72 % of simulated ONT 2D reads (median over 100 randomly selected unique OTUs) could be mapped to the correct reference and 88 % (median) could be mapped to a sequence belonging to the same species as the reference (Fig. 2a). In comparison, corrected synthetic INC-Seq reads with six segments could be mapped to the correct reference and species with median accuracies of 92 % and 99 %, respectively (Fig. 2a).

We next evaluated overall classification performance based on simulated reads from 100 reference sequences and using different similarity thresholds for read assignment. Based on the F1 score (that is, the harmonic mean of precision and recall), synthetic INC-Seq reads provided consistently better performance compared with simulated ONT 2D reads, as well as corrected reads from CANU [11] (Fig. 2b). INC-Seq reads provided consistently high precision (98 % for an identity threshold of >98 %) and good recall (>90 %) even at an identity threshold of 97 % (Additional file 1). CANU corrected reads generally provided high precision but low recall (about 5 %), whereas raw 2D reads typically had high recall but low precision. Of note, these simulations do not involve generating chimeric sequences and may not capture systemic errors in nanopore sequencing and are thus likely to represent easier test cases.

### Assessing chimera rates in INC-Seq libraries with PacBio sequencing

PacBio SMRT sequencing of an INC-Seq 16S rRNA library (hypervariable regions V3 to V6) for a simple synthetic bacterial community (*S. cristatus*, *S. oralis* and *P. micra*; Table 2) was used to estimate chimera rates for INC-Seq and to further evaluate the practicality of this protocol. Raw PacBio reads have a lower mismatch error rate (about 1 %) [12] and can thus facilitate the identification of chimeric constructs. In addition, the median read lengths obtained here were slightly longer than those of MinION reads (4.7 vs 3.6 kbp), although much longer reads can be obtained on the MinION system, allowing a slightly better chimeric read analysis (Additional file 2).

**Table 2:** INC-Seq-estimated abundances for a simple synthetic community

Species	Reference (GenBank ID)	Relative abundance		
		Defined	SMRT	ONT 2D
*Streptococcus oralis*	KT932114.1	0.600	0.552	0.639
*Streptococcus cristatus*	KF933778.1	0.300	0.393	0.309
*Parvimonas micra*	KP944178.1	0.100	0.055	0.052

Using simulated chimeric reads, templates that differed by <20 % could potentially result in a chimeric consensus, if ligated in the correct orientation before RCA (Additional file 3). However, this would be a rare event as the ligation conditions were customized for reduced intermolecular ligation (see ‘Circular ligation and RCA for construction of INC-Seq libraries’). Indeed, by mapping known, distinct reference 16S rRNA amplicon sequences to SMRT reads (see ‘Estimating chimera rates’), the intermolecular ligation rate was estimated to be only 0.06 % (Table 1). Although chimeric consensus sequences can be constructed from similar intermolecularly ligated templates (the divergence is 3 % between *S. cristatus* and *S. oralis*) in the synthetic community, the maximum expected fraction of chimeric consensus sequences was very low (0.036 %; Additional file 3).

The majority of chimeric constructs were estimated to be the result of template switching, with up to 3 % of reads in the SMRT library being attributed to it (Table 1). Such reads could be filtered out by the conservative consensus construction approach, and the expected fraction of such chimeric consensus sequences was even lower (Additional file 3), as the probability that template switching could result in chimeric reads with the correct orientation, as well as concordant segment length, was low (see ‘Anchor-based consensus construction for INC-Seq reads’).

Consensus reads from SMRT INC-Seq were found to cover the full-length amplicon (median: 99 %; mean: 98 %) and improved median identity of SMRT reads from 84 % to 98 % (Additional file 4A,B). Mismatch, insertion and deletion error rates were reduced significantly (one-sided Wilcoxon test *p*-value < 10^-15^; Additional file 4C), and the median total error rate was reduced to below 1 % when more than 15 segment copies were used to construct a consensus (Additional file 4D). The proportion of reads with significantly lower identity than average (2 standard deviations away at 92 %) was low (2.6 %), indicating that few INC-Seq reads are affected by uniformly low accuracy or errors associated with chimeric sequences. Of note, consensus sequences were constructed from called bases, and therefore accuracy is likely to improve further with longer reads and algorithms that analyse the raw signal from the sequencing machine.

### MinION INC-Seq reads provide high-accuracy, full-length 16S amplicon sequences

**Fig. 3:**
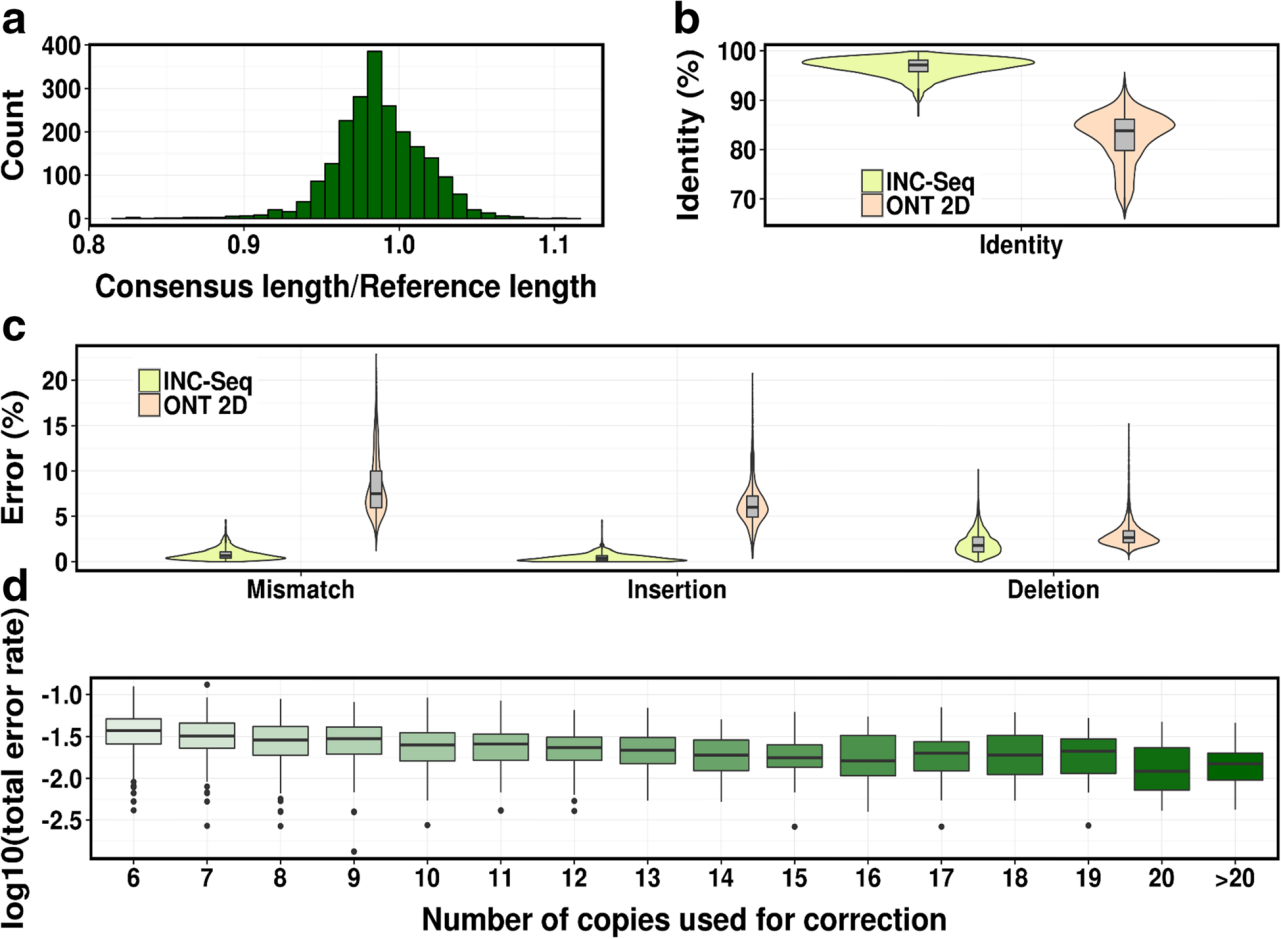
INC-Seq produces long and accurate reads. **a** The ratio between the length of INC-Seq corrected reads and the reference sequence is tightly distributed around 1 (only reads with length from 600-800 bp are shown here). **b**, **c** INC-Seq boosts overall read accuracy and significantly reduces mismatch, insertion and deletion error rates (one-sided Wilcoxon test *p*-value <10^-15^ in all cases). **d** Accuracy of INC-Seq sequences increases with the number of segments used for consensus construction

MinION sequencing of the INC-Seq 16S rRNA library provided similar estimates of intermolecular ligation and template switching rates as was observed for PacBio libraries (Table 1). Consensus sequences were found to span the 16S amplicons (median and mean coverage: 99 %; Fig. 3a), boosting median accuracy of ONT 2D reads from 84 % to 97 % (Fig. 3b). Overall, mismatch rates were reduced tenfold (from 7.5 % to 0.7 %; one-sided Wilcoxon test *p*-value <10^-15^), with significantly improved insertion and deletion error rates (one-sided Wilcoxon test *p*-value <10^-15^; Fig. 3c). Total error rate of consensus reads was found to be negatively correlated with the number of segment copies used to construct the consensus (Spearman ρ = -0.37, *p*-value <10^-15^), but plateaued out after 15 copies (Fig. 3d).

Despite the potential for an amplification bias in INC-Seq, relative abundance estimates were surprisingly close to the defined abundances of the simple community on both sequencing platforms (Table 2). This could partially be explained by the use of long molecules (containing at least six copies of the amplified template) for INC-Seq analysis, which may be less affected by bias owing to exponential amplification. In addition, several long consensus sequences (about 2 kb) were obtained on both sequencing platforms. Many of these were aligned using BLAST to genomic DNA of *streptococci* (92 % identity), indicating that INC-Seq can also successfully capture longer templates in the input.

### INC-Seq enables robust and sensitive 16S rRNA profiling

**Fig. 4:**
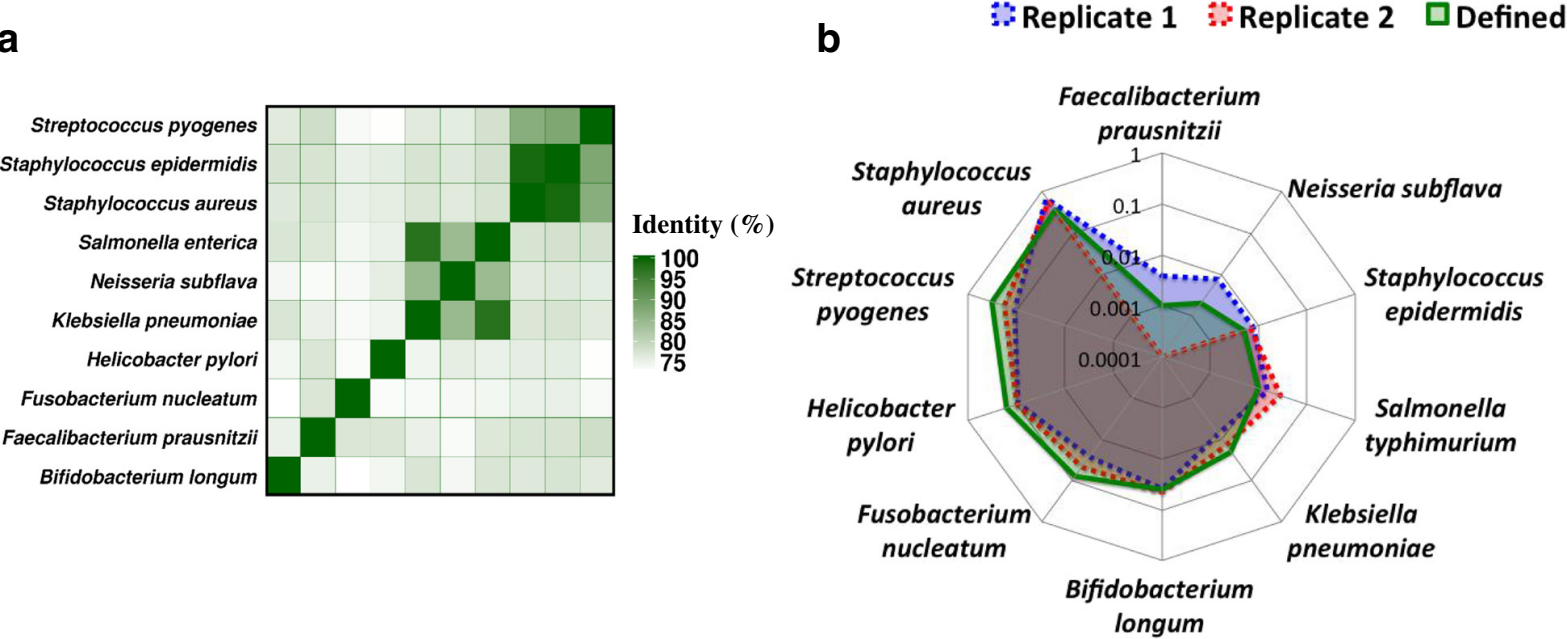
Species detection using INC-Seq. **a** Ten species were selected to construct an artificial community. Some species have highly similar 16S sequences (for example, *S. aureus* and *S. epidermidis* share 99 % identity). **b** Two separate INC-Seq runs produce consistent results that are well correlated with defined abundances (Pearson *ρ* = 0.83, *p*-value = 0.003; Spearman *ρ* = 0.98, *p*-value <10^-15^)

To further evaluate INC-Seq for 16S rRNA profiling, we applied it to a synthetic community of ten microbial species with widely varying relative abundances and in which some bacteria share high similarity (>95 %; Table 3, Fig. 4a). To check reproducibility, two RCA replicates were generated with the same pool of bacterial DNA, and consensus INC-Seq reads were mapped to a comprehensive 16S database with BLAST (see Species profiling using simulated reads). As 16S references can be highly similar, a stringent filter (reference coverage >0.98 and identity >0.98) was applied to remove alignments with low confidence. The resulting abundance profiles were consistent across replicates (Fig. 4b; Pearson ρ = 0.99, *p*-value <10^-8^; Spearman ρ = 0.94, *p*-value <10^-4^) and thus combined for species profiling. In addition, the merged profile was significantly correlated with the defined abundances (Pearson ρ = 0.83, *p*-value = 0.003; Spearman ρ = 0.98, *p*-value <10^-15^). However, relative abundance estimates of the most abundant species in the community were significantly different from defined values, suggesting that INC-Seq libraries of the staggered community might have been more affected by amplification bias, although experimental error in community composition cannot be ruled out. Interestingly, no false positives were detected, except for one read that was ambiguously classified because it was mapped to *Klebsiella pneumoniae* and *Klebsiella variicola* (which differ by one nucleotide) with the same identity (Table 3). Consensus INC-Seq reads can be used to estimate the abundance of *S. epidermidis* at 0.7 % (defined abundance = 0.5 %) despite the presence of *S. aureus* in the community (99 % identical with a defined abundance = 36 %; Fig. 4a). Despite the stringent filters used, INC-Seq can identify all species that are present in the community, including *Faecalibacterium prausnitzii* with a defined relative abundance of 0.1 % (Table 3).

**Table 3:** Species level profiling for a ‘ladder’ synthetic community

Species	Relative abundances	
	Defined	INC-Seq
*Staphylococcus aureus*	0.362	0.631
*Streptococcus pyogenes*	0.320	0.148
*Helicobacter pylori*	0.160	0.096
*Fusobacterium nucleatum*	0.080	0.037
*Bifidobacterium longum*	0.040	0.042
*Klebsiella pneumoniae*	0.020	0.009
*Salmonella typhimurium*	0.010	0.022
*Staphylococcus epidermidis*	0.005	0.007
*Neisseria subflava*	0.002	0.004
*Faecalibacterium prausnitzii*	0.001	0.002

## Discussion and Conclusions

The INC-Seq protocol is relatively easy to implement and thus could potentially be incorporated into automated library preparation workflows. For a modest increase in experimental cost (a few hundred dollars), it provides more accurate long reads on the MinION platform, allowing the use of nanopore sequencing for a wider range of applications. We are currently exploring variations of INC-Seq, including the use of dumbbell adaptors to further refine it and simplify the bioinformatics workflow. Two areas of particular focus are (i) the ability to generate accurate consensus reads of variable-sized fragments without significantly distorting relative abundance estimates and (ii) using longer raw reads to obtain higher consensus read accuracy. The combination of dumbbell adaptors and longer nanopore reads for INC-Seq would enable longer and more accurate reads than are currently feasible with SMRT CCS on the PacBio platform.

We explored the use of INC-Seq for 16S rRNA-based profiling, as it is a widely used approach to investigate complex bacterial communities. Being a targeted approach, it reduces sequencing cost, particularly when non-microbial contamination is high, and is able to capture rare species in a community. Long and accurate INC-Seq reads are well suited to distinguish highly similar 16S sequences, allowing accurate classification at the species level. Amplification of longer regions may improve resolution further [13], but with potential reduction in the set of sequences that can be amplified. Our results demonstrate the potential of INC-Seq in accurately identifying rare species (0.1 % abundance) in a microbial community. Owing to the stringent analysis criteria used, some reads are excluded for being too short or chimeric. However, with increasing read lengths and throughput from nanopore sequencers, the impact on sensitivity may be mitigated. Further experimentation would be necessary to fully understand the limits of detection sensitivity and robustness of INC-Seq with greater sequencing depth [14].

Several steps in the analysis of raw INC-Seq reads could be improved further. The identification of segments to construct the consensus was designed to be conservative but may result in many informative reads and segments being discarded. Segments from chimeric reads could also be used to construct more than one consensus sequence, but we conservatively excluded this option. Median accuracy of INC-Seq reads was improved further (to >98 %) using alternate mappers (GraphMap) to align the segments and by iteratively running PBDAGCON to improve the consensus. In principle, with the use of six or more segment copies, INC-Seq consensus reads should be more accurate than observed, but this is likely to require the development of consensus approaches that realign and analyse sequences at the signal level [15, 16]. With improvements in accuracy of nanopore sequencing from better pores and base-calling algorithms [17], INC-Seq based consensus reads could enable the system to reach parity with accuracy more rapidly on second generation sequencing system (Q20 and Q30) and thus open up even more applications.

Long and accurate reads are key to many genomic applications, such as human haplotype reconstruction, in which polymorphisms are typically separated by a kilobase in the genome. With long and accurate reads, the computational requirement for phasing is greatly reduced [4]. Other applications include exon connectivity detection [3] and viral quasi-species analysis [18, 19]. With the release of PromethION, a highly scalable and flexible real-time sequencing and analysis platform (300× more pores than the MinION system) [20], we anticipate that nanopore sequencing will be cost effective for many of these applications and more practical on the basis of an improved accuracy with INC-Seq.

## Methods

### Bacterial DNA extraction, PCR amplification of 16S rRNA and construction of synthetic communities

Bacterial DNA was obtained from DSMZ Germany for *P. micra* (DSM 20468), *S. cristatus* (DSM 8249), *S. oralis* (DSM 20627), *F. prausnitzii* (DSM 17677), *Neisseria subflava* (DSM 17610), *Bifidobacterium longum* subsp. *longum* (DSM 20219), *Fusobacterium nucleatum* subsp. *polymorphum* (DSM 20482) and *Helicobacter pylori* (DSM 21031). Bacterial cultures were obtained from ATCC (Manassas, VA, USA) for *Staphylococcus epidermidis* (ATCC 12228), *Salmonella typhimurium* subsp. *enterica* (ATCC 13311), *Klebsiella pneumoniae* subsp. *pneumoniae* (ATCC 700721), *Streptococcus pyogenes* (ATCC 49117) and *Staphylococcus aureus* subsp. *aureus* (ATCC 31240). Bacterial cultures were grown in ATCC-recommended media, and DNA was extracted using a genomic DNA isolation kit (bio-52066, Bioline, Taunton, MA, USA), according to manufacturer’s protocol. PCR amplification of 16S rRNA was carried out using previously designed primers [13], 338 F (5’ Phos-ACTYCTACGGRAGGCWGC-3’) and 1061R (5’ Phos-CRRCACGAGCTGACGAC-3’), using Phusion High-Fidelity PCR Master Mix (F531, Thermo Fisher Scientific, Waltham, MA, USA) according to the manufacturer’s instructions. The conditions for PCR were set up as follows: initial denaturation at 98 °C for 30 s, 15 cycles of 98 °C for 10 s with annealing at 59 °C for 30 s and extension at 72 °C for 30 s, and final extension at 72 °C for 5 min. PCR products were purified with Agencourt AMPure XP beads (Beckman Coulter, Indianapolis, IN, USA). Each PCR product was diluted down before being pooled together according to defined relative abundances (using their mass as a proxy to achieve a total mass of 100 ng in 100 μl volume) for each community. Two synthetic bacterial communities were constructed (Tables 2, 3). The ‘simple’ community consists of three bacteria, whereas the ‘ladder’ community consists of ten bacteria with large variation in relative abundances.

### Circular ligation and RCA for construction of INC-Seq libraries

Circular ligation of pooled (by mass) DNA was performed in 3 ml reaction volume with T4 DNA ligase (M0202, New England Biolabs, Ipswich, MA, USA) according to manufacturer’s protocol. The mixture was incubated at 16 °C for 6 h before heat inactivation of T4 DNA ligase at 65 °C for 10 min. Ligated products were purified and concentrated using AMPure XP beads. Plasmid-Safe ATP-Dependent DNase (E3101, Epicentre, Madison, WI, USA) digest was performed according to manufacturer’s protocol at 37 °C for 1 h to remove non-circularized products. DNase was then heat inactivated at 70 °C for 30 min. Products were purified and concentrated using AMPure XP beads. The circular DNA template molecules were added to phi29 DNA polymerase reaction buffer (B0269, New England Biolabs) with 0.25 μg/μl BSA, 5 μM exo-resistant random primers (S0181, Thermo Fisher Scientific), 0.25 mM dNTP (N0447, New England Biolabs) and 10 U phi29 polymerase (M0269, New England Biolabs) in a total volume of 20 μl. The conditions for RCA were set up as follows: 95 °C for 3 min, 30 °C (ramp rate of 0.1 °C/s) for 6 h and 65 °C for 10 min. The time taken for amplification was optimized to yield substantial RCA products, while minimizing the possibility of sequences produced by unspecific priming of random primers. RCA products were finally purified with 0.45× volume of AMPure XP beads.

### PacBio library preparation and sequencing

The RCA product of the simple community was purified and concentrated with 1× volume AMPure® PB Beads (100-265-900, PacBio, Menlo Park, CA, USA) giving 7 μg of purified product, determined by Qubit 2.0 fluorometer (Life Technologies, Carlsbad, CA, USA). The purified product was gently sheared (to reduce hyperbranched DNA structures) using Covaris® g-TUBE™ device (Covaris, Woburn, MA, USA) at 6,000 rpm for 1.5 min. To verify the size of fragments after shearing, sheared and unsheared products were run on a 0.5 % (w/v) agarose gel. The sheared RCA product was purified with 0.45× AMPure® Beads and was used to prepare the 10 kb SMRTbell™ template in accordance with the Pacific Biosciences procedure for ‘10 kb Template Preparation and Sequencing’. BluePippin™ (Sage Science, Beverly, MA, USA) was used to select libraries of sizes ranging from 6-10 kb. The distribution of library sizes was first assessed on Agilent 2100 Bioanalyzer (Agilent Technologies, Inc., Santa Clara, CA, USA) with the DNA 12000 kit (5067-1509, Agilent Technologies, Inc.) before they were sequenced on a PacBio sequencer, using P5-C3 chemistry coupled with MagBead Standard Seq v2 collection protocol.

### Nanopore library preparation and MinION sequencing

RCA DNA products were fragmented using Covaris® g-TUBE™ (Covaris) centrifuged at 3,200 rpm for 3 min. The ONT SQK-MAP006 protocol was followed, with some modifications to the Run B protocol from Urban et al. [21]. Briefly, starting material of 8 μg was used, the RCA products were handled using wide-bore tips and 0.4× AMPure XP beads purification was performed after DNA repair and end prep. As more starting material was used, DNA repair, end prep and adapter ligation were performed in double the volume with double the reagents. All elutions were carried out for 20 min at 37 °C. Samples were processed with SQK-MAP006 kits and run on SQK-MAP006 flow cells on a MinION MK1 device. The raw FAST5 reads were uploaded to the online server for base calling through the Metrichor software (version 1.48). ‘2D pass’ reads in FASTA format were extracted using poretools (version 0.5.1) [22].

### Anchor-based consensus construction for INC-Seq reads

For each original INC-Seq read, subsequences were extracted using non-overlapping sliding windows of 500 base pairs (until half the read length) to serve as ‘anchors’ that is, subsequences 1-500, 501-1,000, 1,001-1,500 and so on). The anchors were aligned to the read with BLASTN (for high sensitivity) [23]. Alignments covering less than 80 % of an anchor were discarded. Anchors with the higher number of alignments were used to extract repeating segments in the read. The starting positions of two adjacent anchors define a candidate repeating segment (Fig. 1b). To extract segments that are as complete as possible, the segment end corresponding to the 5’-end anchor was extended according to the clipped bases of the anchor at its 5’-end. For each read, the longest run of consecutive segments with the same orientation and similar lengths (up to 5 % difference from the median length) was used to construct a consensus sequence. Each segment extracted from a run was tested as a potential backbone by aligning other segments against it with BLASTN. The segment with the higher number of alignments (similar results were sorted out with the average percentage identity of alignments) was used as the final backbone. Reads having less than six aligned segments were discarded. BLASTN alignments were converted to BLASR m5 format and a consensus sequence was constructed using PBDAGCON (-t 2 -c 1 -m 5; git commit #de1cf85) [24].

### Species profiling using simulated reads

A customized 16S reference database for species classification was constructed by trimming sequences in the SILVA database (release 123) [25] to the amplicon regions (for example, from V3 to V6) [13]. References without species level annotation were filtered out, and the database was deduplicated with vsearch (version 1.9.3) [26]. A customized version of NanoSim [27] that allows control for the length of simulated reads was used to simulate ONT 2D reads [28]. To generate appropriate error models from these data, three trimmed reference 16S sequences (*Streptococcus oralis*, *Streptococcus cristatus* and *Parvimonas micra*) were aligned against each read from the simple synthetic community with BLASTN to cut each read into segments according to the alignments. Error models were generated with NanoSim's read_analysis.py script with all segments and the three references as inputs. One hundred operational taxonomic unit (OTU) sequences were randomly selected from the customized reference database, and ONT 2D reads were simulated (using the learnt error models) from each OTU sequence with NanoSim's simulator.py script (the length for simulated reads was set to be similar to the reference). To simulate RCA reads, synthetic amplified sequences were created by concatenating each reference sequence 40 times. Simulated reads were generated using simulator.py script, in which simulated read lengths were determined by the number of copies that are required for error correction.

Overall, the same number of reads was simulated from 100 randomly selected references. CANU (version 1.2) was used to correct all reads using the following parameters: minReadLength = 100, minOverlapLength = 50, corMhapSensitivity = high, corMinCoverage = 2, errorRate = 0.035, corMaxEvidenceErate = 0.3 [11]. To account for loss of reads in the INC-Seq pipeline, we generated 10× number of ONT 2D reads compared to INC-Seq reads. Raw, CANU-corrected and INC-Seq-corrected reads were mapped to the customized database with BLASTN. Species names were assigned with the species annotation of the hit with highest identity, and reads mapped ambiguously to multiple species with the same identity were discarded. Alignments below different identity thresholds (ranging from 0.8 to 0.98) were filtered out, and the list of species detected was compared with the species present in the simulation to calculate the precision, recall and F1 score (that is, the harmonic mean of precision and recall) for classification.

In INC-Seq, chimeras can potentially result from (i) intermolecular ligation of different 16S sequences and (ii) template switching during amplification. To simulate chimeric reads, two random OTUs were selected at varying divergence levels with vsearch. For chimeras from intermolecular ligation, the two references were joined with equal probability in the same or opposite orientations, and the joint template was concatenated 40 times to simulate an RCA product. INC-Seq reads with six copies of the joint template were simulated with NanoSim. To simulate template switching chimeras, INC-Seq reads with >3 copies of the template were simulated from both selected references, and the reads were then concatenated. Consensus reads longer than 1,000 base pairs were filtered out for this analysis as being likely chimeric.

### Estimating chimera rates

To estimate chimera rates, reference 16S sequences were trimmed to the amplicon region and then aligned to PacBio and nanopore reads from the simple community using BLASTN (version 2.2.28+) [29]. Alignments with the lowest *e*-value were selected for each region of the reads, and alignments covering less than 80 % of the 16S query were discarded. As *S. oralis* has 97 % identity to *S. cristatus*, the two *Streptococcus* species were grouped in this analysis.

For intermolecular ligation, reads containing alternating patterns of both references (at least two copies of each) were considered as evidence and the intermolecular ligation rate was estimated to be:

$$ \frac{Number\  of\  reads\  with\  alternating\  references}{Total\  number\  of\  reads\times \left(0.9\times 0.1\times 2\right)} $$

The scaling term (0.9 × 0.1 × 2) accounts for the fact that we only observe intermolecular ligations across genera (the relative abundances of *Streptococci* and *Parvimonas* are 0.9 and 0.1, respectively). Similarly, for template switching, the number of reads containing both references but without an alternating pattern were considered as evidence, and the template switching rate was estimated to be:

$$ \frac{Number\  of\  reads\  with\  both\  genera\  and\  no\  alternating\  pattern}{Total\  number\  of\  reads\times \left(0.9\times 0.1\times 2\right)} $$

### Assessing the quality of corrected sequences

The simple synthetic community was used to estimate the error rates of raw and corrected reads. For raw reads, the three trimmed reference 16S sequences were aligned against each read with BLASTN, and alignments with the lowest *e*-value were selected for each region. Error rate estimates (that is, the percentage of mismatches, insertions and deletions) for each read were determined as the ratio of the total number of errors to the alignment length. For corrected reads, each read was aligned against the three trimmed references, and only the best alignment by *e*-value was kept. The error rate estimates were determined as the ratio of the total number of errors to the total alignment length.

### Species level profiling with INC-Seq corrected reads

INC-Seq corrected reads were self-concatenated (to recover the correct starting and end points) and mapped against the customized SILVA database (see the ‘Species profiling using simulated reads’ section) with BLASTN. Alignments were filtered by reference coverage (>98 %) and percentage identity (>98 %). Species names were assigned according to the hit with highest identity to the query, and ambiguously mapped reads were discarded. The abundance of each reference species was estimated as the proportion of corrected reads that were mapped to that reference.

### Availability and Requirements

- Project name: INC-Seq
- Project home page: https://github.com/CSB5/INCSeq.
- Archived version: DOI: 10.5281/zenodo.57405
- Operating system: Linux.
- Programming language: Python (2.7).
- Other requirements: None.
- License: MIT License.
- Any restrictions to use by non-academics: None.

### Availability of supporting data

Raw sequencing data was uploaded to European Nucleotide Archive (ENA) under project PRJEB12294 (http://www.ebi.ac.uk/ena/data/view/PRJEB12294). Further supporting data can be found in the *GigaScience* repository, GigaDB [30].

## Abbreviations

CCS, circular consensus sequencing; INC-Seq, Intramolecular-ligated Nanopore Consensus Sequencing; ONT, Oxford Nanopore Technologies; RCA, rolling circle amplification; SMRT, single molecule, real-time.

## Additional files

Additional file 1: Read length distribution of SMRT and ONT reads (read length on x-axis and counts on y-axis). (PDF 119 kb) Additional file 2: Fraction of expected chimeric consensus reads (chimeric fraction) due to intermolecular ligation and template switching under different divergence levels between the two template sequences. Each curve shows the average over 100 replicates. (PDF 107 kb) Additional file 3: INC-Seq performance on SMRT reads. (A) The ratio between the length of INC-Seq corrected reads and reference sequences is tightly distributed around 1 (mean: 0.982; standard deviation 0.055). (B, C) INC-Seq boosts overall read accuracy and significantly reduces mismatch, insertion and deletion error rates. (D) Accuracy of INC-Seq sequences increases with the number of segments used for consensus construction. (PDF 101 kb) Additional file 4: Precision and recall curves for detecting 100 species with simulated ONT 2D and INC-Seq reads. ONT 2D reads were also corrected with CANU. Each curve shows the average over 10 replicates. (PDF 147 kb)

## References

[1] Ashton PM, Nair S, Dallman T, Rubino S, Rabsch W, Mwaigwisya S (2014). MinION nanopore sequencing identifies the position and structure of a bacterial antibiotic resistance island. Nat Biotechnol.

[2] Quick J, Loman NJ, Duraffour S, Simpson JT, Severi E, Cowley L (2016). Real-time, portable genome sequencing for Ebola surveillance. Nature.

[3] Bolisetty MT, Rajadinakaran G, Graveley BR (2015). Determining exon connectivity in complex mRNAs by nanopore sequencing. Genome Biol.

[4] Ammar R, Paton TA, Torti D, Shlien A, Bader GD (2015). Long read nanopore sequencing for detection of HLA and CYP2D6 variants and haplotypes. F1000Res.

[5] Benítez-Páez A, Portune KJ, Sanz Y. Species-level resolution of 16S rRNA gene amplicons sequenced through the MinION^TM^ portable nanopore sequencer. Gigascience. 2016;5:4. 10.1186/s13742-016-0111-z PMC4730766 26823973

[6] Loose M, Tyson JR, de Cesare M, Brown BL, Jain M, Ip CLC (2015). Phase 1 data release and analysis. F1000Res.

[7] Hong LZ, Hong S, Wong HT, Aw PPK, Cheng Y, Wilm A (2014). A method for obtaining long viral haplotypes from short sequence reads. Genome Biol.

[8] Travers KJ, Chin C-S, Rank DR, Eid JS, Turner SW (2010). A flexible and efficient template format for circular consensus sequencing and SNP detection. Nucleic Acids Res.

[9] Lou DI, Hussmann JA, McBee RM, Acevedo A, Andino R, Press WH (2013). High-throughput DNA sequencing errors are reduced by orders of magnitude using circle sequencing. Proc Natl Acad Sci U S A.

[10] Acevedo A, Brodsky L, Andino R (2014). Mutational and fitness landscapes of an RNA virus revealed through population sequencing. Nature.

[11] Berlin K, Koren S, Chin C-S, Drake JP, Landolin JM, Phillippy AM (2015). Assembling large genomes with single-molecule sequencing and locality-sensitive hashing. Nat Biotechnol.

[12] Laehnemann D, Borkhardt A, McHardy AC (2015). Denoising DNA deep sequencing data-high-throughput sequencing errors and their correction. Brief Bioinform.

[13] Ong SH, Kukkillaya VU, Wilm A, Lay C, Ho EXP, Low L (2013). Species identification and profiling of complex microbial communities using shotgun Illumina sequencing of 16S rRNA amplicon sequences. PLoS One.

[14] Juul S, Izquierdo F, Hurst A, Dai X, Wright A, Kulesha E, et al. What’s in my pot? Real-time species identification on the MinION. bioRxiv. 2015; doi:10.1101/030742.

[15] Loman NJ, Quick J, Simpson JT (2015). A complete bacterial genome assembled de novo using only nanopore sequencing data. Nat Methods.

[16] Szalay T, Golovchenko JA (2015). De novo sequencing and variant calling with nanopores using PoreSeq. Nat Biotechnol.

[17] Brown C. “No Thanks, I’ve Already Got One”. https://github.com/lmmx/talk-transcripts/blob/master/Nanopore/NoThanksIveAlreadyGotOne.md. Accessed 28 June 2016.

[18] Zagordi O, Bhattacharya A, Eriksson N, Beerenwinkel N (2011). ShoRAH: estimating the genetic diversity of a mixed sample from next-generation sequencing data. BMC Bioinformatics.

[19] Wilm A, Aw PPK, Bertrand D, Yeo GHT, Ong SH, Wong CH (2012). A sequence-quality aware, ultra-sensitive variant caller for uncovering cell-population heterogeneity from high-throughput sequencing datasets. Nucleic Acids Res.

[20] PromethION - Products & services - Oxford Nanopore Technologies. https://www.nanoporetech.com/products-services/promethion. Accessed 11 July 2016.

[21] Urban JM, Bliss J, Lawrence CE, Gerbi SA. Sequencing ultra-long DNA molecules with the Oxford Nanopore MinION. bioRxiv. 2015; doi:10.1101/019281.

[22] Loman NJ, Quinlan AR (2014). Poretools: a toolkit for analyzing nanopore sequence data. Bioinformatics.

[23] Sović I, Šikić M, Wilm A, Fenlon SN, Chen S, Nagarajan N (2016). Fast and sensitive mapping of nanopore sequencing reads with GraphMap. Nat Commun.

[24] PacificBiosciences/pbdagcon. GitHub. https://github.com/PacificBiosciences/pbdagcon. Accessed 11 July 2016.

[25] Quast C, Pruesse E, Yilmaz P, Gerken J, Schweer T, Yarza P (2013). Improved data processing and web-based tools. Nucleic Acids Res.

[26] torognes/vsearch. GitHub. https://github.com/torognes/vsearch. Accessed 11 July 2016.

[27] Yang C, Chu J, Warren RL, Birol I. NanoSim: Nanopore Sequence Read Simulator Based on Statistical Characterization. bioRxiv. 2016; doi:10.1101/044545. 10.1093/gigascience/gix010 PMC5530317 28327957

[28] CSB5/NanoSim. GitHub. https://github.com/CSB5/NanoSim. Accessed 11 July 2016.

[29] Altschul SF, Gish W, Miller W, Myers EW, Lipman DJ (1990). Basic local alignment search tool. J Mol Biol.

[30] Li C, Chng KR, Boey EJH, Ng AHQ, Wilm A, Nagarajan N. Supporting data for “INC-Seq: accurate single molecule reads using nanopore sequencing”. GigaScience Database. 2016. doi:10.5524/100208. 10.1186/s13742-016-0140-7 PMC4970289 27485345

