# Supplementary figures and images for: INC-Seq: accurate single molecule reads using nanopore sequencing
Source: Gigascience. 2016 Aug 2;5:34. doi: 10.1186/s13742-016-0140-7 (PMC4970289; doi:10.1186/s13742-016-0140-7)

Read length distribution of SMRT and ONT reads (read length on x-axis and counts on y-axis).

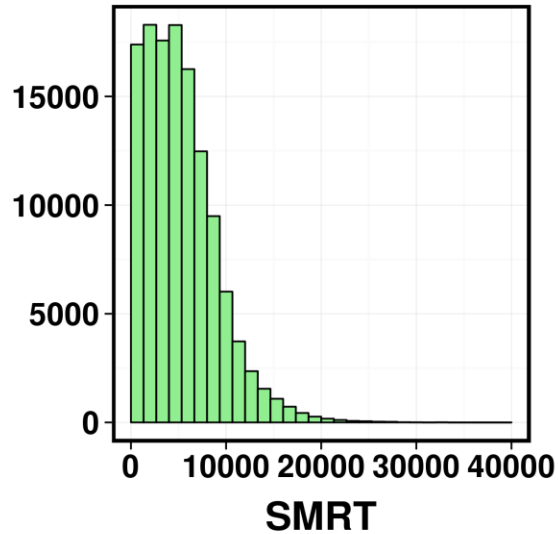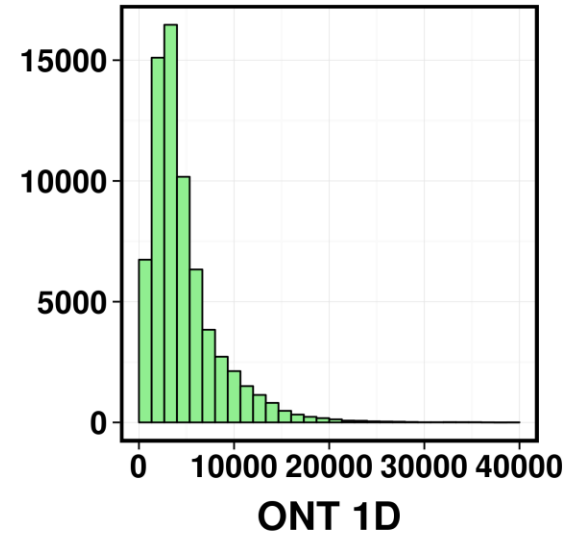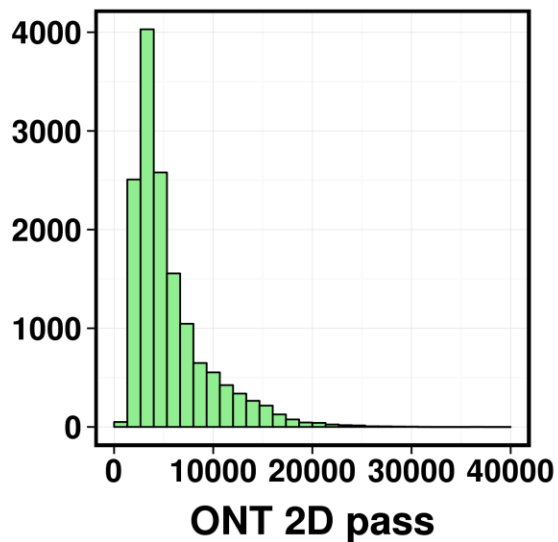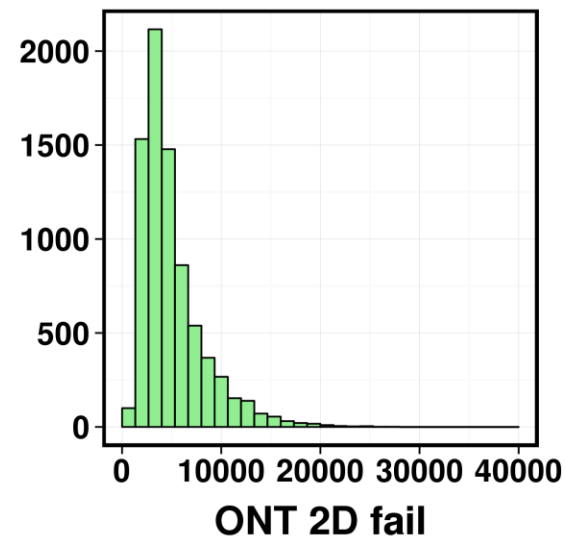

Supplement: Additional file 1: — Read length distribution of SMRT and ONT reads (read length on x-axis and counts on y-axis). (PDF 119 kb) [file 13742_2016_140_MOESM1_ESM.pdf]
