# Supplementary material for: INC-Seq: accurate single molecule reads using nanopore sequencing
Source: Gigascience. 2016 Aug 2;5:34. doi: 10.1186/s13742-016-0140-7 (PMC4970289; doi:10.1186/s13742-016-0140-7)

Fraction of expected chimeric consensus reads (chimeric fraction) due to intermolecular ligation and template switching under different divergence levels between the two template sequences. Each curve shows the average over 100 replicates.

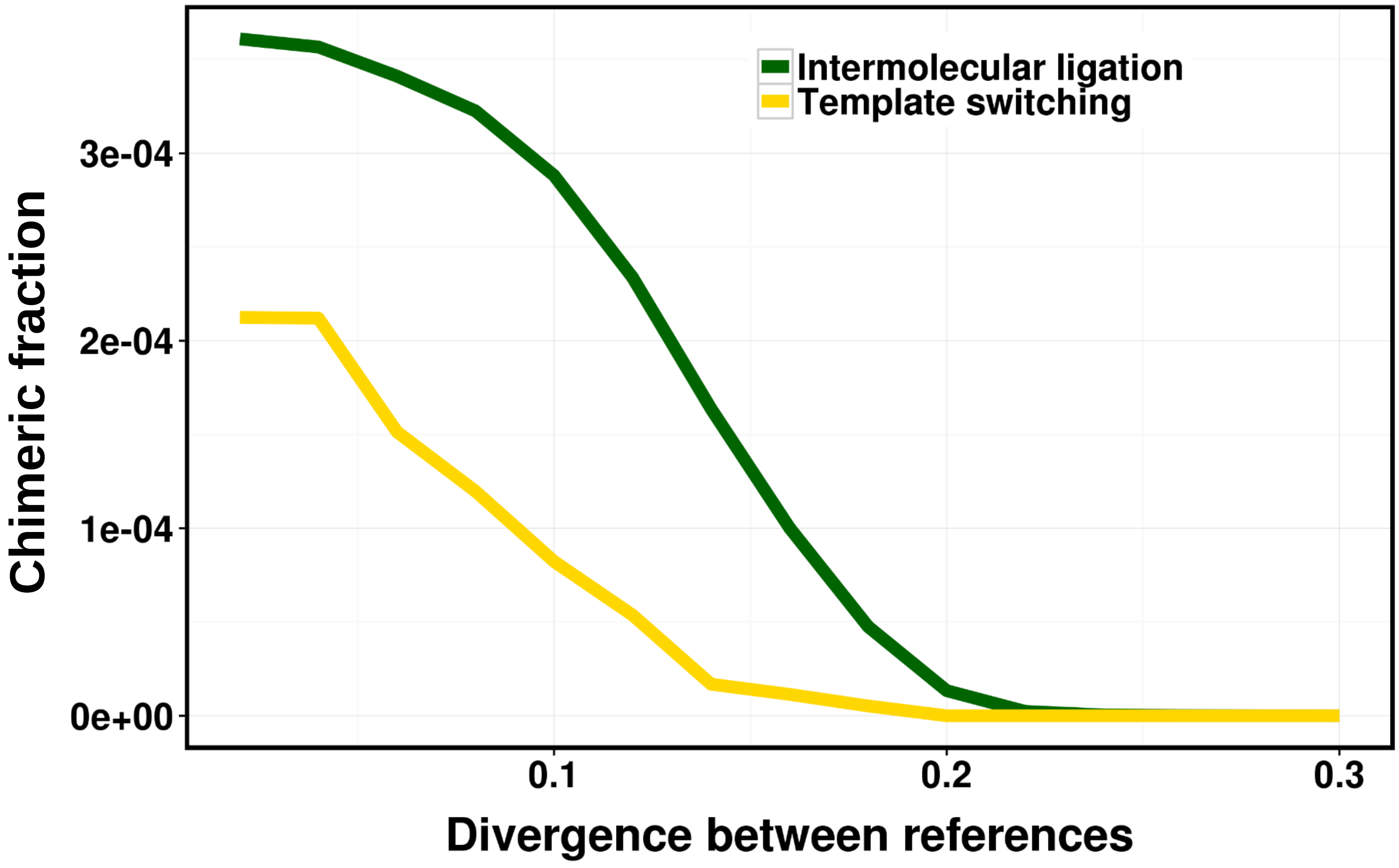

Supplement: Additional file 2: — Fraction of expected chimeric consensus reads (chimeric fraction) due to intermolecular ligation and template switching under different divergence levels between the two template sequences. Each curve shows the average over 100 replicates. (PDF 107 kb) [file 13742_2016_140_MOESM2_ESM.pdf]
