# Supplementary material for: INC-Seq: accurate single molecule reads using nanopore sequencing
Source: Gigascience. 2016 Aug 2;5:34. doi: 10.1186/s13742-016-0140-7 (PMC4970289; doi:10.1186/s13742-016-0140-7)

INC-Seq performance on SMRT reads. (A) The ratio between the length of INC-Seq corrected reads and reference sequences is tightly distributed around one (mean: 0.982; standard deviation 0.055). (B and C) INC-Seq boosts overall read accuracy and reduces mismatch, insertion and deletion error rates significantly. (D) Accuracy of INC-Seq sequences increases with the number of segments used for consensus construction.

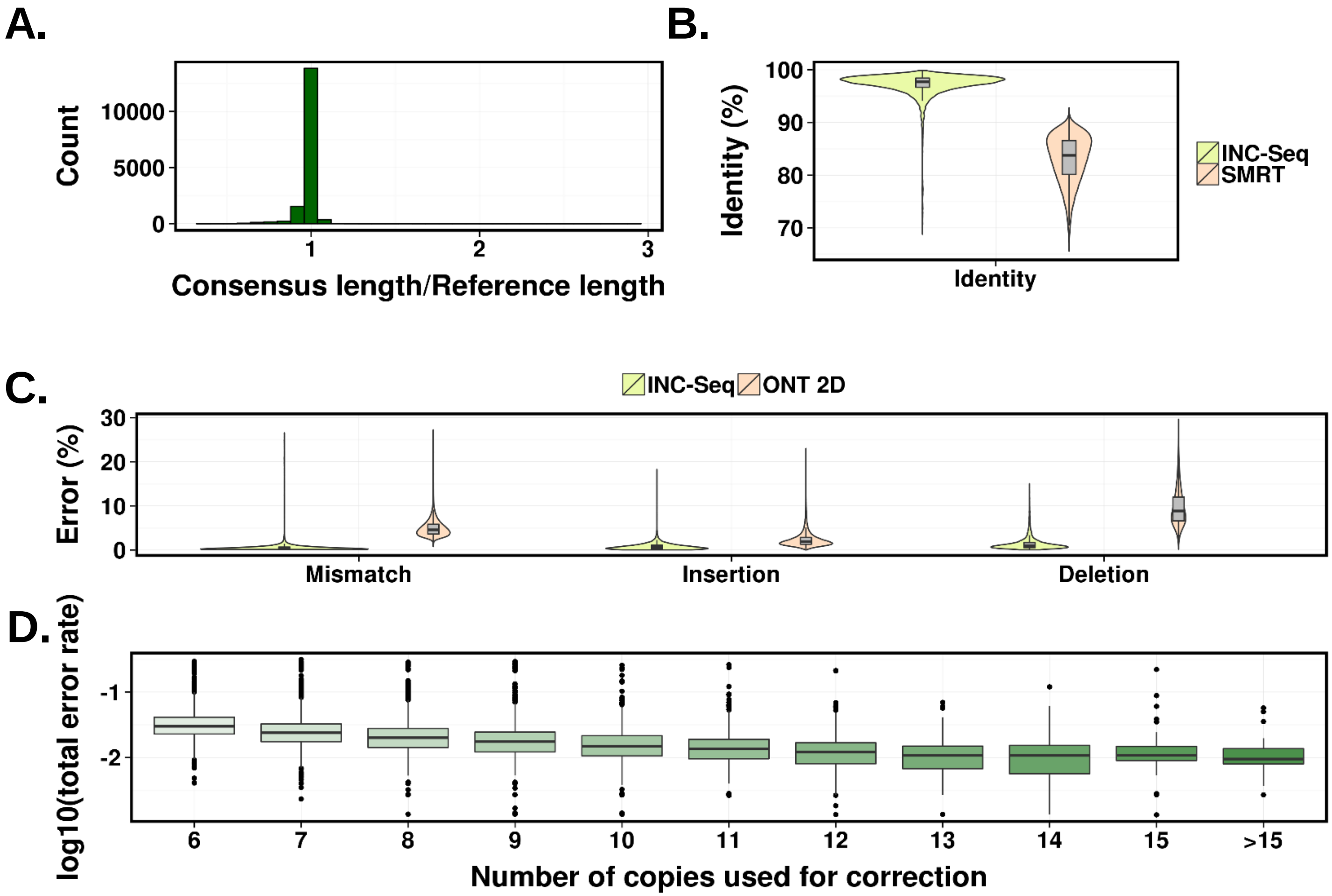

Supplement: Additional file 3: — INC-Seq performance on SMRT reads. (A) The ratio between the length of INC-Seq corrected reads and reference sequences is tightly distributed around 1 (mean: 0.982; standard deviation 0.055). (B, C) INC-Seq boosts overall read accuracy and significantly reduces mismatch, insertion and deletion error rates. (D) Accuracy of INC-Seq sequences increases with the number of segments used for consensus construction. (PDF 101 kb) [file 13742_2016_140_MOESM3_ESM.pdf]
