# Supplementary material for: INC-Seq: accurate single molecule reads using nanopore sequencing
Source: Gigascience. 2016 Aug 2;5:34. doi: 10.1186/s13742-016-0140-7 (PMC4970289; doi:10.1186/s13742-016-0140-7)

Precision and recall curves for detecting 100 species with simulated ONT 2D and INC-Seq reads. ONT 2D reads were also corrected with CANU. Each curve shows the average over 10 replicates.

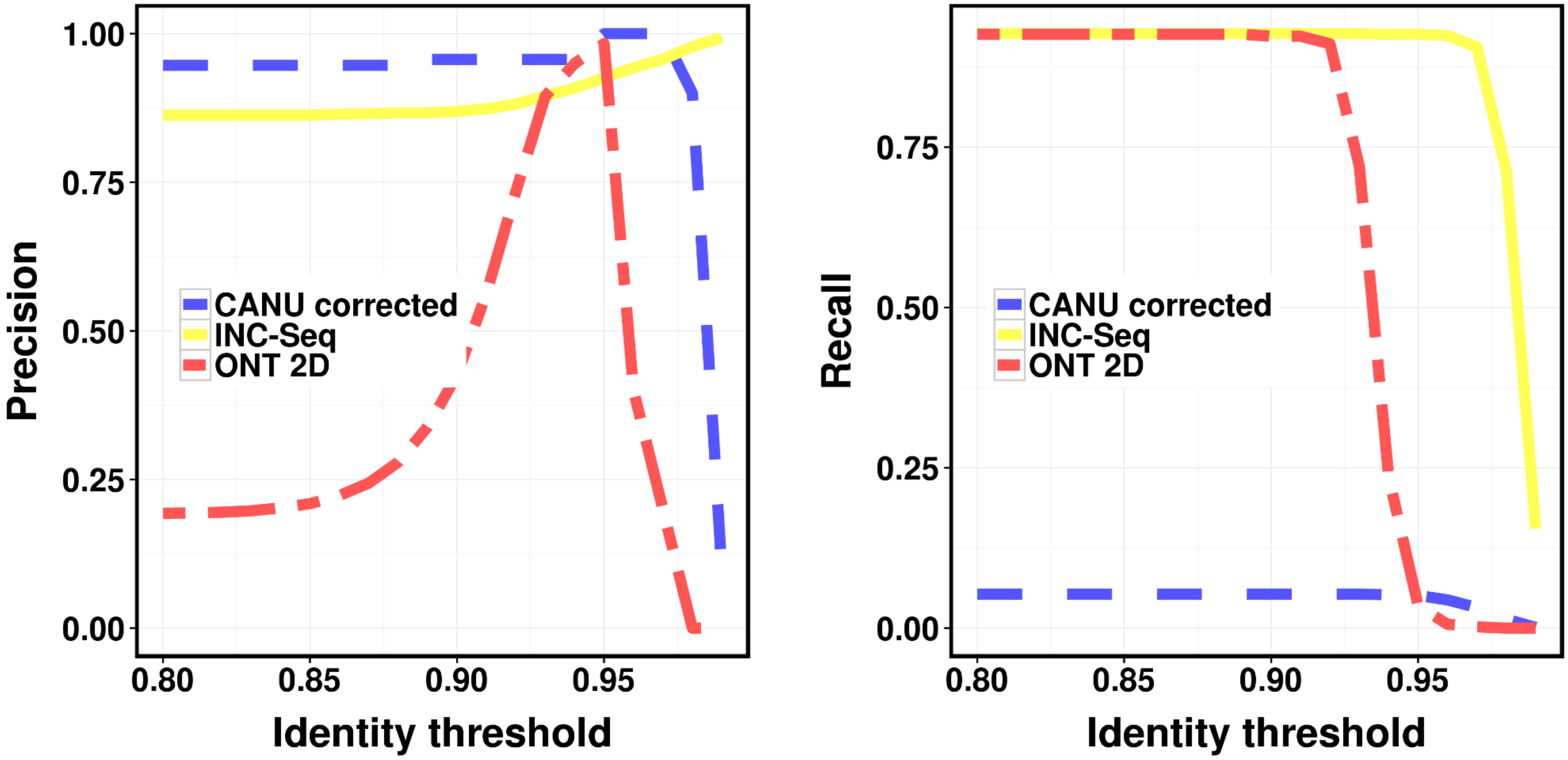

Supplement: Additional file 4: — Precision and recall curves for detecting 100 species with simulated ONT 2D and INC-Seq reads. ONT 2D reads were also corrected with CANU. Each curve shows the average over 10 replicates. (PDF 147 kb) [file 13742_2016_140_MOESM4_ESM.pdf]
